# Scaffolding LSD1 Inhibitors Impair NK Cell Metabolism and Cytotoxic Function Through Depletion of Glutathione

**DOI:** 10.3389/fimmu.2020.02196

**Published:** 2020-09-17

**Authors:** Cavan P. Bailey, Mary Figueroa, Achintyan Gangadharan, Dean A. Lee, Joya Chandra

**Affiliations:** ^1^Department of Pediatrics—Research, MD Anderson Cancer Center, Houston, TX, United States; ^2^Department of Epigenetics and Molecular Carcinogenesis, MD Anderson Cancer Center, Houston, TX, United States; ^3^The University of Texas MD Anderson Cancer Center UTHealth Graduate School of Biomedical Sciences, Houston, TX, United States; ^4^Department of Pediatrics, Nationwide Children's and the Ohio State Comprehensive Cancer Center, Columbus, OH, United States

**Keywords:** LSD1, NK cell, antioxidant, metabolism, glutathione

## Abstract

Cell therapies such as chimeric-antigen receptor (CAR) T-cells and NK cells are cutting-edge methods for treating cancer and other diseases. There is high interest in optimizing drug treatment regimens to best work together with emerging cell therapies, such as targeting epigenetic enzymes to stimulate recognition of tumor cells by immune cells. Herein, we uncover new mechanisms of the histone demethylase LSD1, and various inhibitors targeting unique domains of LSD1, in the function of NK cells grown for cell therapy. Catalytic inhibitors (tranylcypromine and the structural derivatives GSK LSD1 and RN-1) can irreversibly block the demethylase activity of LSD1, while scaffolding inhibitors (SP-2509 and clinical successor SP-2577, also known as seclidemstat) disrupt epigenetic complexes that include LSD1. Relevant combinations of LSD1 inhibitors with cell therapy infusions and immune checkpoint blockade have shown efficacy in pre-clinical solid tumor models, reinforcing a need to understand how these drugs would impact T- and NK cells. We find that scaffolding LSD1 inhibitors potently reduce oxidative phosphorylation and glycolysis of NK cells, and higher doses induce mitochondrial reactive oxygen species and depletion of the antioxidant glutathione. These effects are unique to scaffolding inhibitors compared to catalytic, to NK cells compared to T-cells, and importantly, can fully ablate the lytic capacity of NK cells. Supplementation with biologically achievable levels of glutathione rescues NK cell cytolytic function but not NK cell metabolism. Our results suggest glutathione supplementation may reverse NK cell activity suppression in patients treated with seclidemstat.

## Introduction

Cellular therapies are rapidly being investigated for applications in infectious disease, autoimmunity, and oncology. Numerous clinical trials are testing the combination of infused cell therapies with targeted therapies, including small molecules and antibodies, with the aim of increasing efficacy of the cell product at the disease site. Epigenetic drugs targeting chromatin modifiers are among these potential combinations, with available agents for a range of targets including acetylated histone readers (BETs), histone deacetylases, methyltransferases, and demethylases (1). The histone H3K4 demethylase LSD1 has been investigated as a target in Ewing sarcoma and AML, where LSD1 inhibition induces differentiation of AML cells (2) and blocks fusion protein transcriptional targets in sarcoma (3). Among tumors with low mutational burdens, it has been proposed that epigenetic inhibitors can make these cancers more visible to the immune system by activating gene expression programs (4). Recently, it has been demonstrated that LSD1 inhibition can accomplish this by stimulating T-cell immunity in epithelial cancers (5, 6) and innate immunity in pediatric brain tumors (7).

Available LSD1 inhibitors operate through two distinct binding mechanisms: irreversible catalytic site inhibitors and reversible scaffolding inhibitors. Both types of inhibitors can block the demethylase function, but scaffolding inhibitors also interfere with LSD1 in complex with other epigenetic regulators (8). LSD1 presence is critical for normal hematopoietic development in the terminal erythroid and megakaryocytic compartments (9, 10), but there remains little information on the effects of LSD1 inhibitors directly on mature cytotoxic T- and NK cells. In a combination treatment scheme, small molecule LSD1 inhibitors will also encounter infused immune cells in peripheral blood and the local tumor microenvironment. Ergo, it is crucial to understand how LSD1 inhibitors of differing potencies and binding mechanisms may affect T- and NK cells. Epigenetic regulation of NK cells by chromatin modifiers has previously been linked to methyltransferase EZH2 (11, 12), demethylases KDM5A (13), and JMJD3 (14), and the deubiquitinase MYSM1 (15). Notably, Cribbs et al. (14) included a small molecule epigenetic compound screen for IFN-gamma secretion from NK cells, but only catalytic LSD1 inhibitors (TCP and GSK LSD1) were included at low doses (20 and 0.5 μM, respectively).

Our group has previously published that the scaffolding LSD1 inhibitor SP-2509 and its clinical successor SP-2577, or seclidemstat, potently suppress the viability and metabolism of NK cells (7). The NK cells in our previous report and this current study are expanded from healthy human donors into a cell therapy-grade product. This strategy has been applied in clinical trials for oncology and hematopoietic stem cell transplant (16, 17). LSD1 has previously been implicated in metabolic regulation in adipose tissue (18) and red blood cells (19), but we were the first to show this effect in NK cells. In this report, we further expand upon our previous findings to uncover an induced oxidative stress response that is unique to NK cells, compared to T-cells, and unique to scaffolding LSD1 inhibitors compared to catalytic inhibitors. We are the first to link LSD1 to redox response in NK cells, and we further delineate the critical role of glutathione (GSH) in NK cell cytotoxic response. Importantly, since GSH can be regulated systemically in humans by dietary supplementation, our data raises the possibility of a simple and safe intervention to prevent NK cell dysfunction in patients treated with seclidemstat.

## Materials and Methods

### Primary Human Samples and Cell Line Culture

Human *ex vivo* expanded NK cells were previously isolated from de-identified healthy donor peripheral blood mononuclear cells (PBMCs), expanded with feeder cells, and cryopreserved as stocks in liquid N_2_ (20). Expanded NK cells were cultured in RPMI (Corning) supplemented with 10% FBS (Genesee Scientific) + 1% of each of the following: penicillin/streptomycin (HyClone), NEAA (Lonza), L-glutamine (Sigma), sodium pyruvate (Lonza), and HEPES (ThermoFisher). One-hundred units per milliliter IL-2 was added to NK cultures every 3 days as needed. Human T-cells were isolated from healthy donor PBMCs using the EasySep Human T-cell Isolation Kit, cultured in ImmunoCult-XF T-cell Expansion Medium, and stimulated to grow with ImmunoCult Human CD3/CD28/CD2 T Cell Activator supplemented with 100 U/mL IL-2 (all from StemCell Technologies). MOLM13 and K562 cells were cultured in the same media as NK cells but without IL-2.

### Chemicals and Reagents

LSD1 inhibitors tranylcypromine (TCP) (Enzo Biosciences), GSK LSD1 (Cayman Chemical), RN-1 (Cayman Chemical), SP-2509 (Cayman Chemical), and SP-2577 (kindly provided by Salarius Pharmaceuticals) were reconstituted in DMSO or PBS (TCP) and aliquoted for storage at −20°C. Glutathione ethyl ester (GSHee) (Cayman Chemical) was suspended in PBS and aliquoted at −20°C. Trolox (Cayman Chemical) and mitoquinol (MQ) (Cayman Chemical) were suspended in DMSO and aliquoted at −20°C. SKQ1 (Cayman Chemical) was provided in a 1:1 EtOH:H_2_O solution and diluted in cell culture media for experiments. Calcein AM (Cayman Chemical) was resuspended in DMSO and aliquoted at −20°C.

### Antibodies and Dyes for Flow Cytometry

Antibodies were used at manufacturer recommended concentrations and cells were incubated at 4°C for 25 mins prior to washing and acquisition: CD3 FITC (BD Biosciences), CD56 PE (BD Biosciences), CD16 PE-Cy7 (ThermoFisher), SLAMF7 PE (BioLegend), and NKG2D APC (ThermoFisher). Ghost Dyes Red 780 and Violet 450 (Tonbo Biosciences) were diluted 1:9 (Red 780) and 1:4 (Violet 450) for use in 50 μL PBS/sample to stain cells for 10 mins at RT before addition of antibodies or other dyes. Monochlorobimane (mBCL) (Sigma) was used at 20 μM in PBS to stain cells for 20 mins at 37°C and acquired in the AmCyan channel. MitoSOX Red (ThermoFisher) was used at 1 μM in PBS to stain cells for 20 mins at 37°C and acquired in the PE channel. MitoTracker Deep Red (ThermoFisher) was used at 250 nM in PBS to stain cells for 20 mins at 37°C and acquired in the APC channel. Cells were washed with FACS buffer (PBS + 2% BSA + 0.01% sodium azide) and resuspended in 300°μL FACS buffer for acquisition on a Fortessa flow cytometer (BD Biosciences) with 405/488/640 nm laser setup. Compensation was calculated using FACSDiva software and UltraComp beads (ThermoFisher) stained with indicated antibodies.

### Cellular Metabolic Analysis

NK and T-cells were pre-treated with indicated compounds for 48 h, counted on a ViCell XR analyzer (Beckman Coulter), washed in PBS, and resuspended in Seahorse XF base DMEM (Agilent) supplemented with 10 mM glucose (Sigma), 2 mM L-glutamine, and 1 mM sodium pyruvate. CellTak (Corning) was used to adhere 300,000 live cells per well in a Seahorse 96-well-plate (Agilent). XF Mito Stress Test kit (Agilent) was used with 1 μM oligomycin, 0.5 μM FCCP, and 0.5 μM rotenone/antimycin A with the standard injection protocol. Analysis was performed on a Seahorse XFe96 analyzer (Agilent) using Wave 2.6.1 software.

### Cytotoxicity Co-culture

NK cells were pre-treated for 48 h with LSD1 inhibitors (±2.5 mM GSHee), counted on a ViCell XR analyzer, washed in PBS, and resuspended at 2 × 10^6^ live cells/mL in supplemented RPMI. Cells were plated in a round-bottom 96-well-plate in 100 μL/well and serially diluted once to make 10:1 and 5:1 effector-to-target ratios in triplicate. Background wells were loaded with 100 μL media only and maximum release wells were loaded with 100 μL 2% Triton-X in media. K562 cells were counted and resuspended at 1 × 10^6^ live cells/mL and incubated with 5 μM calcein AM for 1 h at 37°C with mixing every 10 mins. After calcein AM loading, cells were washed in PBS, counted, and resuspended at 2 × 10^5^ live cells/mL and 100 μL was added to each well of the plate. After centrifugation at 100 × g for 2 mins, the plate was incubated at 37°C for 4 h. After incubation, wells were gently mixed to distribute released calcein AM and the plate was centrifuged at 400 × g for 2 mins. 100 μL of supernatant was transferred to a black opaque flat-bottom 96-well-plate (Nunc) and fluorescence was read on a Synergy 2 plate reader (BioTek) with 485 nm excitation/528 nm emission filter set. Percent specific lysis was calculated by the formula: specific lysis = [(experimental release – background release)/(maximum release – background release)] × 100.

### Statistical Analysis and Graphing Software

All statistical tests are *t*-tests with multiple comparisons corrected using false discovery rate (FDR) cutoff set to 1%. GraphPad Prism 8.4.1 was used to make all graphs and run statistical tests.

## Results

**Figure 1:**
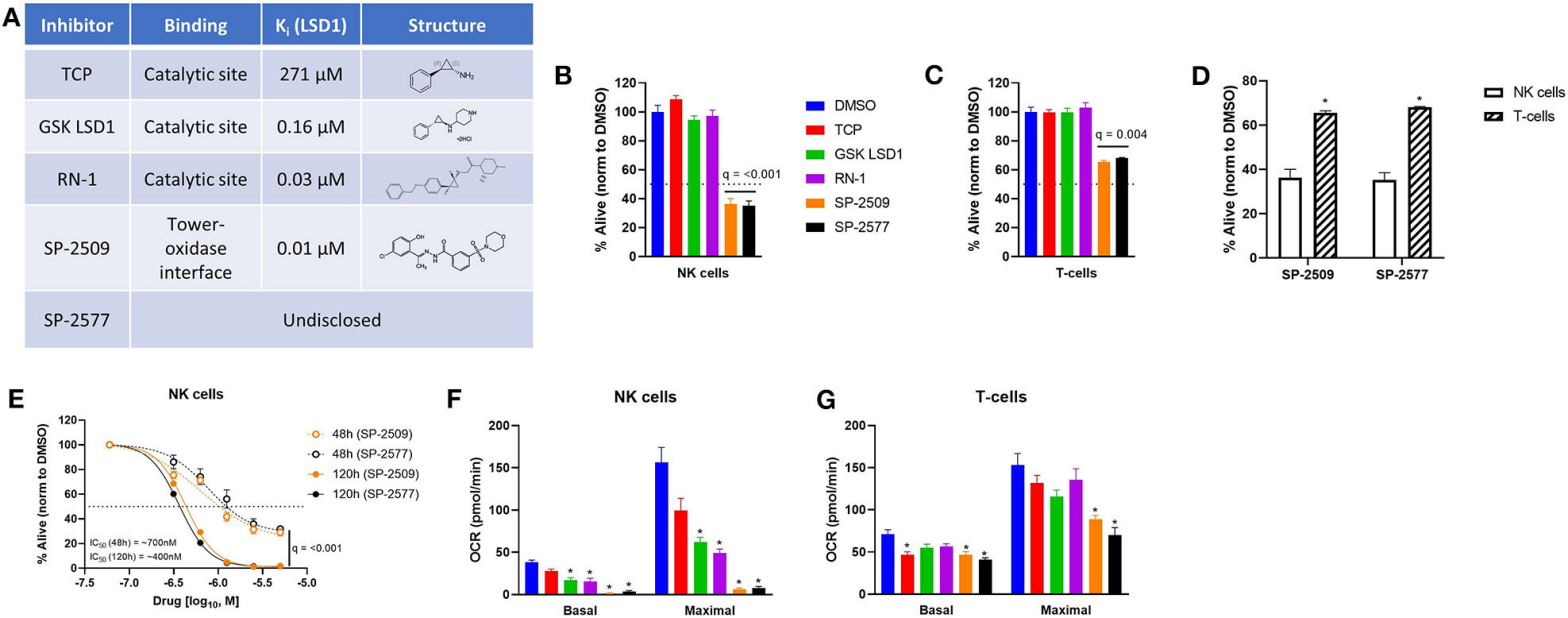
Scaffolding LSD1 inhibitors reduce viability and suppress metabolism in NK cells. **(A)** LSD1 inhibitors used and their respective properties. **(B)** Viability of NK cells after 48 h treatment of LSD1 inhibitors (TCP: 1 mM, GSK LSD1: 100 μM, RN-1: 25 μM, SP-2509: 5 μM, SP-2577: 5 μM) using amine-reactive viability dye analyzed *via* flow cytometry. **(C)** Viability of T-cells using the same method. **(D)** Viability of NK and T-cells under SP-2509 and SP-2577 treatment. *q < 0.01 comparing NK to T-cells *via* unpaired *t*-test. **(E)** Dose response of SP-2509 and SP-2577 in NK cells at indicated time points using amine-reactive viability dye. **(F)** Basal and maximal OXPHOS of NK cells after 48 h treatment with indicated LSD1 inhibitors measured using XF Mito Stress Test on a Seahorse XFe96 analyzer. **(G)** Basal and maximal OXPHOS of T-cells using the same method. *q < 0.01. All conditions are compared to DMSO control *via t*-test with FDR correction. At least three independent experiments are displayed (±SEM), sourced from two unique NK cell donors and 1 T-cell donor.

LSD1 inhibitors can bind to different sites of the LSD1 protein and elicit unique phenotypes in cells. Irreversible catalytic inhibitors TCP, GSK LSD1, and RN-1 form covalent adducts in the demethylation site of LSD1 and block LSD1 activity on histones and other target proteins (Figure 1A). Scaffolding inhibitor SP-2509 acts through a potential allosteric mechanism (21) and can disrupt LSD1 in complex with CoREST (8) in addition to the demethylation function (Figure 1A). We previously observed that scaffolding LSD1 inhibitors were more potent against NK cells compared to T-cells, into the nanomolar range for NK cells, using the AlamarBlue assay (7). Here we replicated doses of LSD1 inhibitors we previously characterized to induce NK reactivity in pediatric brain tumors without exerting direct tumor cytotoxicity, as a model of co-administration, and measured viability using amine-reactive dyes and flow cytometry. Catalytic inhibitors did not reduce viability of NK cells (*q* = n.s.), but scaffolding inhibitors were notably potent at doses 5-200X lower than catalytic inhibitors (Figure 1B, *q* < 0.001). T-cell viability also was reduced by scaffolding LSD1 inhibitors, but were far less sensitive than NK cells (Figure 1C, *q* = 0.004 and Figure 1D, *q* < 0.01). We next examined if NK cell sensitivity to scaffolding LSD1 inhibitors was dose and time dependent, and we found higher doses and longer incubation times amplified the cytotoxic effect (Figure 1E, *q* < 0.001). Our previous publication also found metabolic suppression unique to scaffolding LSD1 inhibitors in NK cells (7). We were able to replicate these findings using numerous unique NK cell donors, observing that scaffolding LSD1 inhibitors abolish all oxidative phosphorylation in NK cells (Figure 1F, ^*^q < 0.01) and reduce OXPHOS to a much lesser degree in T-cells (Figure 1G, ^*^q < 0.01).

**Figure 2:**
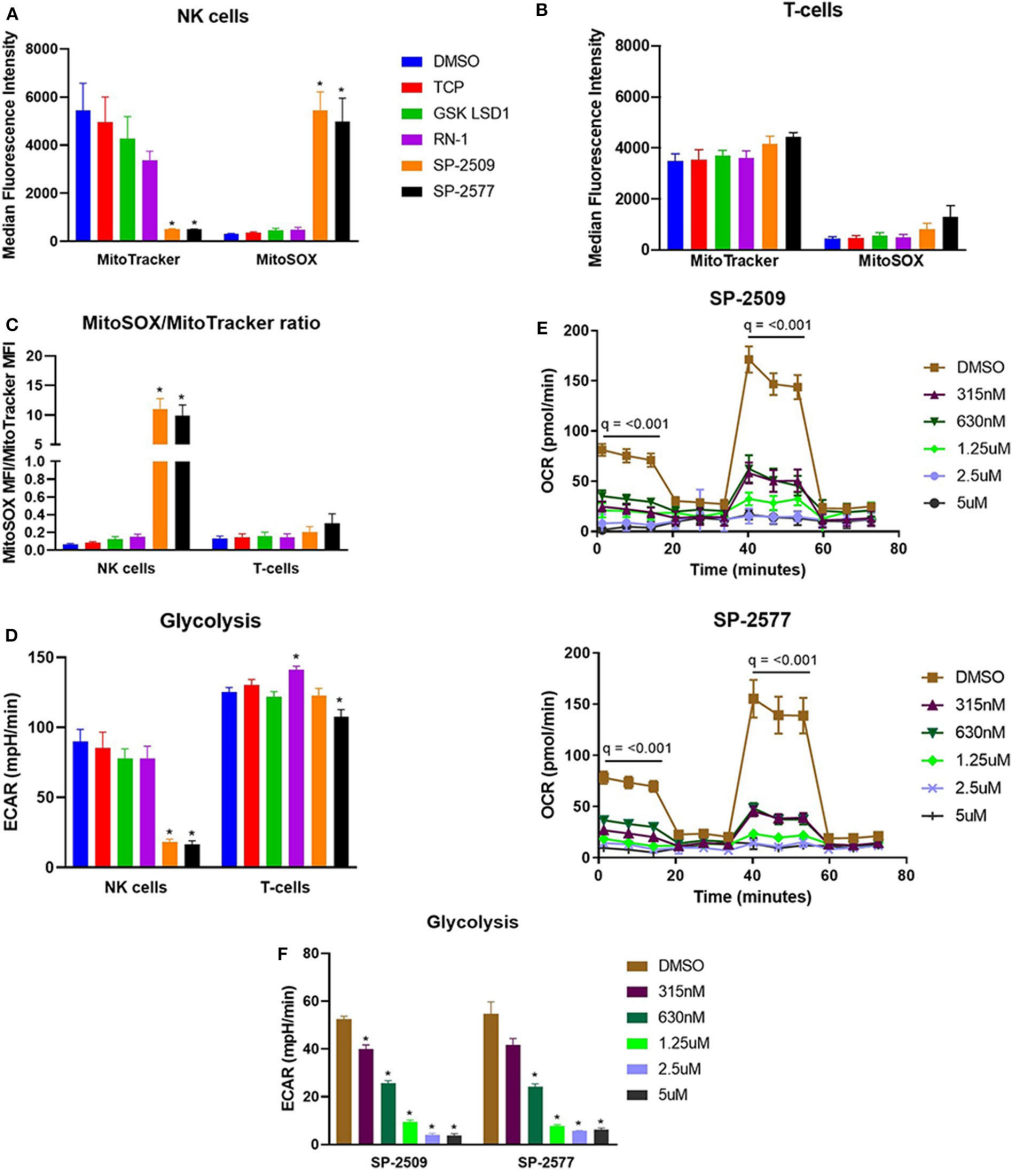
NK cells produce uncontrolled mitochondrial superoxide when treated with scaffolding LSD1 inhibitors. **(A)** NK cells treated for 48 h with indicated LSD1 inhibitors were stained with MitoTracker Deep Red and MitoSOX Red combined with viability dye. Median fluorescent intensity (MFI) of APC channel (MitoTracker) and PE channel (MitoSOX) are plotted from live cells only. **(B)** T-cell MitoTracker and MitoSOX data using the same method. **(C)** NK and T-cell MitoSOX MFI divided by MitoTracker MFI indicates mitochondrial superoxide relative to healthy mitochondria number. **(D)** Basal glycolysis of NK and T-cells treated for 48 h with LSD1 inhibitors measured using XF Mito Stress Test. **(E)** OCR dose response of SP-2509 and SP-2577 in NK cells treated for 48 h and measured using XF Mito Stress Test. **(F)** Basal glycolysis dose response of SP-2509 and SP-2577 in NK cells treated for 48 h and measured using XF Mito Stress Test. *q < 0.01. All conditions are compared to DMSO control *via t*-test with FDR correction. Marked Seahorse data points indicate all treatment conditions are significant vs. DMSO control. At least three independent experiments are displayed (± SEM), sourced from 2 unique NK cell donors and 1 T-cell donor.

Given the extreme mitochondrial dysfunction induced by scaffolding LSD1 inhibitors, we used other molecular probes to examine mitochondrial health in NK cells. Under scaffolding but not catalytic LSD1 inhibitor treatment, we observed a potent drop in healthy mitochondria (MitoTracker) and rise in mitochondrial superoxide production (MitoSOX) in NK cells (Figure 2A, ^*^q < 0.01). Notably, this effect could not be replicated in T-cells (Figure 2B, *q* = n.s.). When normalized to number of healthy mitochondria, superoxide production was over 30× higher in NK cells compared to T-cells under scaffolding LSD1 inhibitor treatment (Figure 2C, ^*^q < 0.01). Interestingly, glycolysis was also reduced only in NK cells under scaffolding LSD1 inhibitor treatment, therefore metabolic effects of this compound class are not limited to mitochondria (Figure 2D, ^*^q < 0.01). We next investigated if drops in oxidative phosphorylation were dose dependent with SP-2509 and SP-2577, and we found even low doses (~315 nM for 48 h) could significantly reduce basal and maximal respiration in NK cells (Figure 2E, *q* < 0.001). However, glycolysis reduction was dose dependent under SP-2509 and SP-2577 treatment (Figure 2F, ^*^q < 0.01).

**Figure 3:**
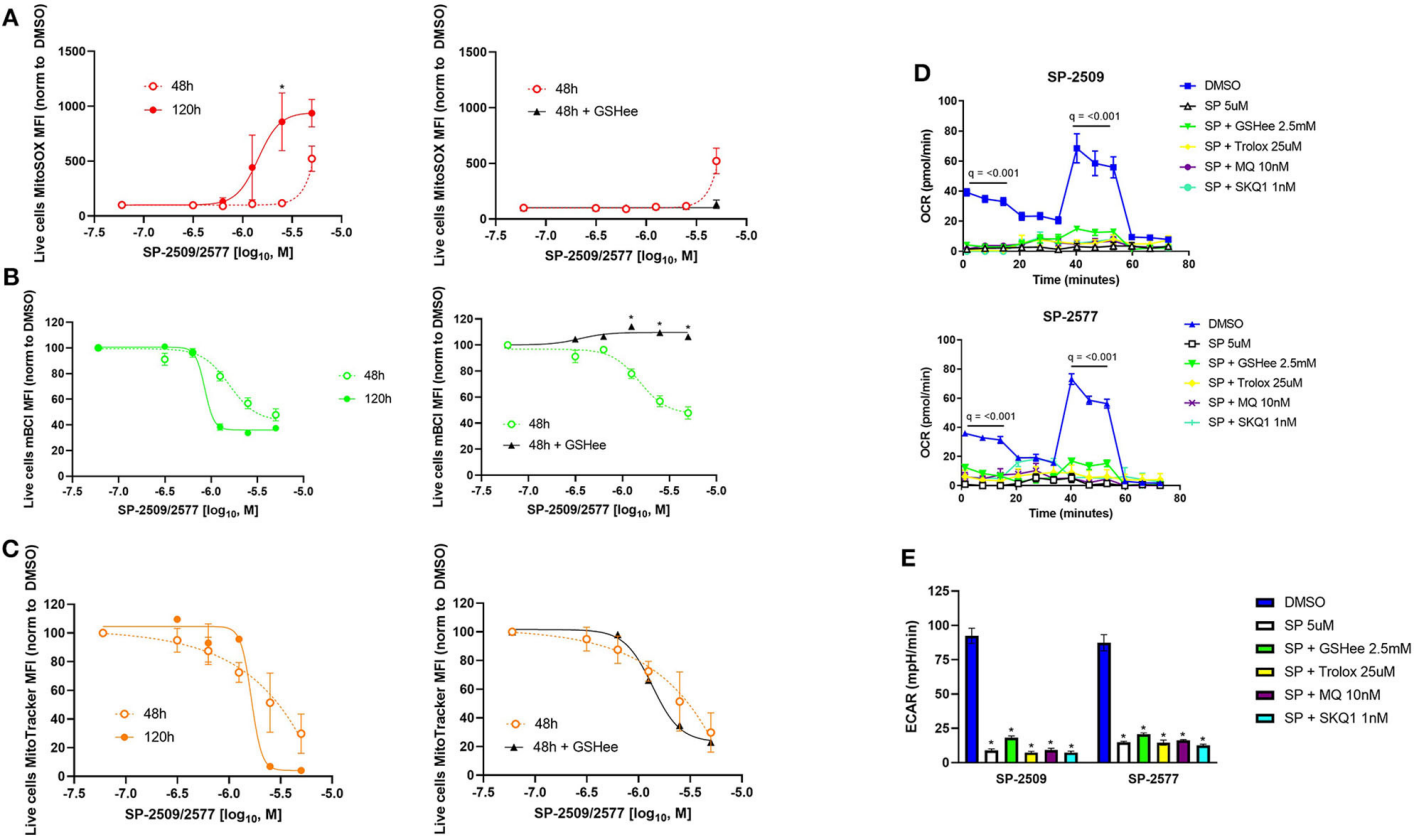
Scaffolding LSD1 inhibitor-induced oxidative stress in NK cells is dose dependent and can be rescued with glutathione supplementation, but metabolism defects cannot. **(A)** MitoSOX dose response of SP-2509 and SP-2577 in live NK cells at indicated time points and rescued using 2.5 mM glutathione ethyl ester (GSHee). **(B)** Glutathione dose response of SP-2509 and SP-2577 in live NK cells measured using mBCL and rescued using 2.5 mM GSHee. **(C)** MitoTracker dose response of SP-2509 and SP-2577 in live NK cells and attempted rescued using 2.5 mM GSHee. **(D)** OXPHOS of NK cells treated with scaffolding LSD1 inhibitors for 48 h and attempted rescue with cell-wide antioxidants (GSHee and Trolox) and mitochondrial-targeted antioxidants [mitoquinol (MQ) and SKQ1] measured using XF Mito Stress Test. **(E)** Basal glycolysis of NK cells treated with scaffolding LSD1 inhibitors for 48 h using the same method and measured using XF Mito Stress test. *q < 0.01. All conditions are compared to DMSO control *via t*-test with FDR correction. Marked Seahorse data points indicate all treatment conditions are significant vs. DMSO control. At least three independent experiments are displayed (± SEM), sourced from two unique NK cell donors.

While mitochondrial function was not dose dependent in NK cells, but viability was, we performed dose responses examining mitochondrial number, superoxide production, and glutathione levels in NK cells treated with SP-2509 and SP-2577. We found superoxide production was time and dose dependent, but this could be decreased by co-supplementation with exogenous glutathione (Figure 3A, ^*^q < 0.05). Treatment with scaffolding LSD1 inhibitors reduced glutathione in a dose dependent manner, potentially explaining the uncontrolled mitochondrial superoxide levels. Here, we also showed that glutathione co-supplementation blocks this reduction with SP-2509 and SP-2577 treatment (Figure 3B, ^*^q < 0.05). Mitochondrial number was also dose dependent, but interestingly not variable by time or glutathione supplementation, suggesting a rapid and oxidative stress-independent mechanism of mitochondrial damage by SP-2509 and SP-2577 (Figure 3C, *q* = n.s.). We next attempted to rescue mitochondrial oxidative phosphorylation and glycolysis by co-supplementation with antioxidants, both cell-wide (2.5 mM GSH and 25 μM Trolox) and mitochondrial-targeted [10 nM mitoquinol (MQ) and 1 nM SKQ1] (22, 23). We found that none of the antioxidants could restore mitochondrial function (Figure 3D, *q* < 0.001) or glycolysis (Figure 3E, ^*^q < 0.05), further suggesting metabolic defects caused by SP-2509 and SP-2577 are not linked to reactive oxygen species (ROS).

**Figure 4:**
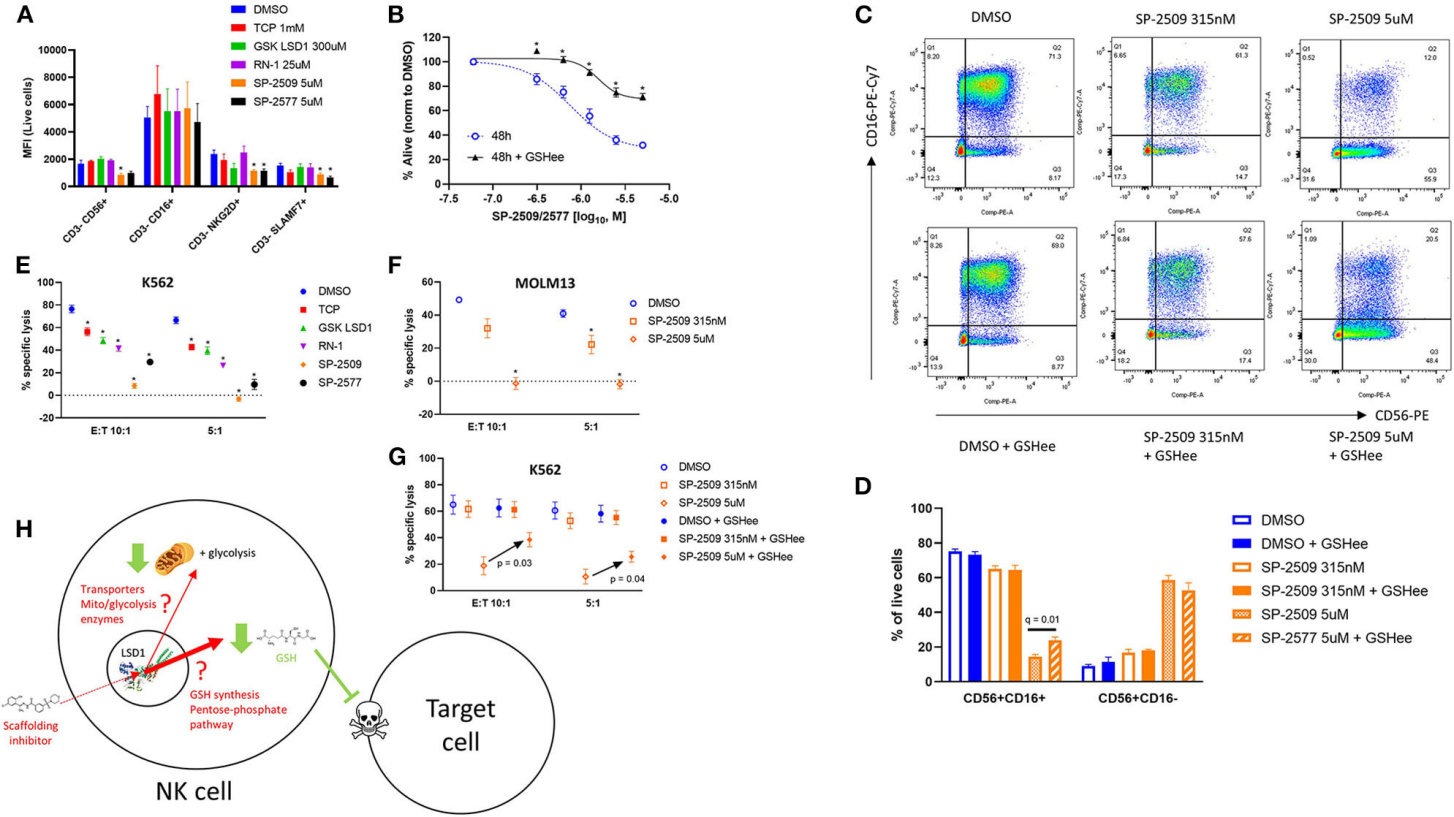
NK cell ligand expression and cytotoxicity are impaired by scaffolding LSD1 inhibitors, but viability and cytotoxicity can be rescued with glutathione supplementation. **(A)** NK cells treated for 48 h with indicated LSD1 inhibitors display reduced activating ligand expression. **(B)** Viability dose response of SP-2509 and SP-2577 in NK cells treated with and without 2.5 mM GSHee supplementation. **(C)** Flow cytometry plots of NK cells treated with SP-2509 at indicated doses for 48 h, with and without 2.5 mM GSHee supplementation. **(D)** Quantification of flow cytometry gates from panel C across three unique NK donors. **(E)** NK cell cytotoxicity against K562 target cells is reduced by 48 h pre-treatment with indicated LSD1 inhibitors. **(F)** NK cell cytotoxicity against MOLM13 target cells after 48 h pre-treatment with SP-2509. **(G)** NK cell cytotoxicity against K562 target cells after 48 h pre-treatment with SP-2509, with and without 2.5 mM GSHee supplementation. **(H)** Working model of scaffolding LSD1 inhibitor effects on NK cell metabolism, redox state, and function. *q < 0.01. All conditions are compared to DMSO control *via t*-test with FDR correction. At least three independent experiments are displayed (± SEM), sourced from three unique NK cell donors.

We next evaluated functional determinants of NK cell biology, primarily their receptor phenotype and ability to lyse target cells. Multicolor flow cytometry revealed that only scaffolding LSD1 inhibitors reduce activating receptors NKG2D and SLAMF7 expression on NK cells (Figure 4A, ^*^q < 0.05). We hypothesized that glutathione co-supplementation could restore NK function, and indeed we found that viability was rescued by GSH (Figure 4B, ^*^q < 0.05). We further observed that SP-2509 causes NK cells to lose CD16 in a dose-dependent manner, and that GSH supplementation could prevent some CD16 loss (Figures 4C,D, *q* = 0.01). Next, we co-incubated LSD1 inhibitor pre-treated NK cells with labeled K562 target cells to assess their cytotoxic function. Here, we observed that all LSD1 inhibitors reduced NK lysis ability, with SP-2509 and SP-2577 being by far the most potent (Figure 4E, ^*^q < 0.05). The SP-2509 lytic suppression effect was recapitulated with another target cell, the acute leukemia line, MOLM13, and furthermore was dose-dependent (Figure 4F). Glutathione co-supplementation was able to rescue the cytotoxicity defect at high doses of SP-2509 (Figure 4G). Our proposed model of scaffolding LSD1 inhibitors in NK cells is metabolic suppression at low doses and an independent pro-oxidative induction at high doses that potently blunts NK cell cytotoxic function (Figure 4H).

## Discussion

Our report is the first to show therapeutic inhibition of LSD1 *via* scaffolding inhibitors initiates functionally relevant pro-oxidative effects in NK cells. While the direct mechanism of LSD1 redox regulation in NK cells remains to be discovered, LSD1 has been linked to cellular oxidative stress in two previous reports. In studies of macrophage resistance to hydrogen peroxide, Tokarz et al. (24) found that inhibiting LSD1 with SP-2509 increases cell viability and reduces superoxide, the opposite of our observations. The mechanism in macrophages was driven by short lived (<9 h) enhancement of *SOD2* transcription by reversal of demethylation of H3K4me2 induced by LPS stimulation. They did not compare SP-2509 to other LSD1 inhibitors with catalytic binding nor did they examine glutathione levels. Their findings demonstrate the lineage-specific effects of scaffolding LSD1 inhibitors, as we observed between NK and T-cells. Mishra et al. (25) observed that silencing of LSD1 with siRNA in retinal endothelial cells increased H3K4me1/H3K4me2 at the promoter of *GCLC*, the key enzyme in GSH synthesis that binds glutamate to cysteine. They also saw increased binding of NRF2 at the *GCLC* promoter under LSD1 siRNA along with increased *GCLC* expression. This is again opposed to our observation of LSD1 preserving glutathione levels, however no LSD1 inhibitors were used in their investigation so it cannot be said if binding sites on LSD1 play a role or if the differential response is due to tissue type. Another possible explanation for GSH loss under LSD1 inhibition is downregulation of glucose transporters, which has previously been observed with LSD1 knockdown (26). Reductions in glucose import would dampen the pentose-phosphate pathway, leading to reduced NADPH production and an inability to recharge GSH from its oxidized GSSG form. Potential NK cell dependence on cystine importer *SLC7A11* expression would make them sensitive to glucose deprivation *via* disulfide accumulation, already a noted vulnerability in cancer cells (27, 28). The above findings may be potential mechanisms connecting LSD1 to glutathione in NK cells, but we are the first to observe key differences using a thorough suite of catalytic and scaffolding LSD1 inhibitors.

GSH has been previously demonstrated to play an important role in immune cell function, including detailed mechanisms in T-cells and correlative nutritional studies in NK cells. Kurniawan et al. (29) recently reported an elegant mouse model of GSH-deficiency in regulatory T-cells, where GSH controls serine metabolism through *ASCT1* expression and subsequently activates mTOR/SMAD3/FoxP3 signaling to endow T_regs_ with their suppressive capabilities. GSH was also shown to be critical for cytotoxic T-cell responses *via* a NFAT-dependent glycolysis mechanism (30), but this has been found to be dispensable in NK cells (31). Mitochondrial metabolism has also been suggested to be critical to NK function. Intratumoral hypoxia was shown to promote tumor escape from innate immunity potentially by suppression of NK OXPHOS (32), and fatty acid uptake by NK cells in obese patients reduced their OXPHOS and lytic function (33). Herein, we have shown NK cells maintain high cytotoxicity despite markedly suppressed mitochondrial OXPHOS, and that GSH can rescue cytotoxic function independently of oxygen consumption or lactic acid production. Notably, neither of these reports investigated glutathione or oxidative stress, but an earlier report found obese mice had defective leukocyte lysis and lowered GSH levels (34). This phenotype could be rescued by adding eicosapentaenoic and docosahexaenoic acids to the diet, suggesting dietary interventions can be used to combat immune cell oxidative stress.

Given our findings that scaffolding LSD1 inhibition depletes GSH and blunts NK activity, it may be possible for oral supplementation of GSH or its precursors to be combined with LSD1 inhibitors in patients. A previous report showed cysteine and theanine supplementation can boost NK cytotoxicity in humans, but the authors did not measure glutathione levels despite cysteine being the rate-limiting amino acid in GSH synthesis (35). In other human trials, oral GSH supplements could boost cytotoxicity against K562 cells (36, 37), and low glutathione in blood tracked with low cytotoxicity in autistic patients (38), however, these studies differ from ours in that whole PBMCs were used for the cytotoxicity assays instead of isolated NK cells. *In vitro* NK functions can also be augmented against infectious *M. tuberculosis* (39), and rescued after treatment by tri/dibutylin (40) or reactive nitrogen metabolites (41), by GSH supplementation. The natural compound adenanthin produces similar cytotoxicity defects and ROS accumulation in NK cells at similar concentrations to scaffolding LSD1 inhibitors (42). While adenanthin does not deplete glutathione nearly as potently as SP-2509 or SP-2577, NK cell cytotoxicity could be rescued with N-acetylcysteine which can replenish GSH levels. Adenanthin is a proposed peroxiredoxin 1 (PRDX1) inhibitor, which reduces hydrogen peroxides and alkyl hydroperoxides, and may be a downstream mediator of our LSD1 inhibitor effect on GSH loss and cytotoxicity suppression (43).

Adding to the above previous knowledge, our data highlights the crucial role of GSH in innate immune responses and defines a new role for LSD1 and potential complex members in maintaining NK cell redox status. The tower domain of LSD1 and its interactions with CoREST may play a mechanistic role in this phenomenon, given that catalytic LSD1 inhibition does not phenocopy scaffolding LSD1 inhibition. RNA-Seq data shows that expanded NK cells maintain expression of LSD1, CoREST, HDAC1, HDAC2, and GFI1 compared to naïve NK cells from the same donor (manuscript in preparation), indicating the LSD1 complex may remain important for NK cell oxidative balance in patients treated with LSD1 inhibitors. The number and function of NK cells in patients in seclidemstat trials (NCT03895684, NCT03600649) has so far not been reported. It remains unknown if *in vivo* pharmacokinetics (half-life, *C*_max_) may limit the seclidemstat NK cell toxicity we have observed. Even so, because glutathione is a safe and readily available supplement it may be advisable to include it in future seclidemstat trials, particularly if NK cell activity is expected to contribute to anti-tumor efficacy. Our data encourages incorporation of oxidative stress and metabolic parameters in immune cells, particularly NK cells, as investigative endpoints.in future investigations of LSD1 inhibition.

## Data Availability Statement

The raw data supporting the conclusions of this article will be made available by the authors, without undue reservation.

## Author Contributions

Performed experiments: CB, MF, and AG. Provided research materials and edited the manuscript: DL. Conceived hypotheses, designed experiments, wrote, and edited the manuscript: CB, MF, and JC. All authors: contributed to the article and approved the submitted version.

## Conflict of Interest

The authors declare that the research was conducted in the absence of any commercial or financial relationships that could be construed as a potential conflict of interest.
